# Adverse pregnancy outcomes associated with moderate elevations in blood pressure or blood glucose in Ugandan women; a prospective cohort study

**DOI:** 10.1016/j.xagr.2021.100007

**Published:** 2021-03-04

**Authors:** Jack Milln, Betty Nakabuye, Barnabas Natamba, Isaac Sekitoleko, Michael Mubiru, Arthur Namara, Samuel Tumwesigire, Tino Salome, Mandy Mirembe, Ayoub Kakanda, Brian Agaba, Faridah Nansubuga, Daniel Zaake, Ben Ayiko, Herbert Kalema, Sarah Nakubulwa, Musa Sekikubo, Annettee Nakimuli, Emily L. Webb, Moffat J. Nyirenda

**Affiliations:** a Non-Communicable Diseases Theme, Medical Research Council/Uganda Virus Research Institute and London School of Hygiene and Tropical Medicine (MRC/UVRI & LSHTM) Uganda Research Unit, Entebbe, Uganda (Drs Milln, Natamba, and Nyirenda and Mr Sekitoleko, Mubiru, Namara, Tumwesigire, Ms Salome, Mirembe, and Mr Kakanda); b Department of Endocrinology and Diabetes, Queen Mary University of London, London, United Kingdom (Dr Milln); c Department of Obstetrics and Gynaecology Uganda Martyrs Hospital, Lubaga, Kampala, Uganda (Drs Nakabuye and Agaba); d School of Public Health, Makerere University College of Health Sciences, Kampala, Uganda (Dr Nakabuye); e Department of Obstetrics and Gynaecology, St. Francis Hospital, Nsambya, Kampala, Uganda (Drs Nansubuga and Zaake); f Department of Obstetrics and Gynaecology, Entebbe Regional Referral Hospital, Entebbe, Uganda (Dr Ayiko); g Department of Obstetrics and Gynaecology, Masaka Regional Referral Hospital, Masaka, Uganda (Dr Kalema); h Department of Obstetrics and Gynaecology, School of Medicine, Makerere University College of Health Sciences, Kampala, Uganda (Drs Nakubulwa, Sekikubo and Nakimuli); i Department of Obstetrics and Gynaecology, Kawempe National Referral Hospital, Kampala, Uganda (Drs Nakubulwa, Sekikubo and Nakimuli); j London School of Hygiene and Tropical Medicine (LSHTM), London, United Kingdom (Drs Webb and Nyirenda)

**Keywords:** Africa, gestational diabetes, hypertension, large birthweight, noncommunicable disease, perinatal mortality, pregnancy, Uganda

## Abstract

**BACKGROUND:**

The association between overt hypertension and diabetes and adverse pregnancy outcomes is well documented. Recent evidence suggests that even moderate elevations in blood pressure or blood glucose may confer a significant risk in a dose-dependent manner. However, these studies have primarily been undertaken in white populations in high-income settings. Hypertension and diabetes are emerging as major public health issues in sub-Saharan Africa as the region undergoes rapid urbanization. It is therefore important to understand how such noncommunicable conditions contribute to pregnancy outcomes in these populations.

**OBJECTIVE:**

This study aimed to determine the association between stage 1 hypertension or fasting blood glucose in the gestational diabetes mellitus-range and adverse pregnancy outcomes in Uganda, and to describe the effects of other contributing factors such as maternal obesity.

**STUDY DESIGN:**

This was a prospective cohort study of 2857 women at 5 major hospitals in urban and semiurban central Uganda. Women were enrolled at 24 to 28 weeks’ gestation. Data about the maternal demographics, anthropometrics, fasting venous blood glucose, blood pressure, and pregnancy outcomes were collected. Moderate elevations in blood pressure and blood glucose were defined using the latest American College of Cardiology and American Heart Association definition of stage 1 hypertension and the World Health Organization's criteria for fasting blood glucose in the gestational diabetes mellitus-range. The primary outcomes of interest were perinatal death and large birthweight for gestational age, and the secondary outcomes were preterm birth, cesarean delivery, and neonatal admission. A multivariable logistic regression analysis was used.

**RESULTS:**

Stage 1 hypertension increased the odds of perinatal death by more than 2-fold (adjusted odds ratio, 2.68; 95% confidence interval, 1.36–5.29), with a positive but insignificant association with preterm birth. Hyperglycemia in the gestational diabetes mellitus-range was associated with cesarean delivery only (adjusted odds ratio, 1.65; 95% confidence interval, 1.20–2.27). Maternal obesity increased the risk of having large birthweight babies (adjusted odds ratio, 2.30; 95% confidence interval, 1.74–3.02), a cesarean delivery (adjusted odds ratio, 2.75; 95% confidence interval, 2.17–3.48), and neonatal admission (adjusted odds ratio, 1.63; 95% confidence interval, 1.16–2.30).

**CONCLUSION:**

Moderate elevations in blood pressure and maternal obesity are stronger predictors of adverse maternal and neonatal outcomes than moderate elevations in blood glucose levels and should be the focus of intervention in these resource-poor settings. Further research is needed to determine the cost-effectiveness of identifying and managing moderate elevations in blood pressure and maternal obesity.

**AJOG MFM at a Glance**

**Why was this study conducted?**

Hypertension and diabetes are emerging public health issues in sub-Saharan Africa. This study aimed to determine the association between stage 1 hypertension or mild fasting hyperglycemia and adverse pregnancy outcomes in Africa.

**Key findings**

Stage 1 hypertension increased the odds of perinatal death by almost 3-fold. Although fasting blood glucose levels in the gestational diabetes mellitus-range was associated with a positive trend for large birthweight babies, this was clearly exceeded by the contribution of maternal obesity.

**What does this add to what is known?**

Moderate elevations in blood pressure and maternal obesity are stronger predictors of adverse maternal and neonatal outcomes than moderate elevations in blood glucose levels. These should be the focus of intervention and management in the resource-limited setting.

## Introduction

Hypertensive and hyperglycemic disorders of pregnancy are 2 of the most common pregnancy complications associated with both short- and long-term adverse maternal and neonatal outcomes. The association between poor outcomes and severe forms of these disorders, such as chronic hypertension and diabetes in pregnancy (DIP), is clear.^1^^,^^2^ However, the contribution of less-severe elevations in blood pressure or milder levels of hyperglycemia in the gestational diabetes mellitus (GDM) range remains debatable.

Recent evidence suggests that there may be a graded risk across the spectrum of hypertension or hyperglycemia. For example, there is now widespread recognition of a continuous association between blood pressure and cardiovascular risk. This has led to further categorization into normal, elevated (systolic blood pressure [SBP], 120–129 mm Hg; diastolic blood pressure [DBP], <80 mm Hg), stage 1 (SBP, 130–139 mm Hg; DBP, 80–89 mm Hg), and stage 2 (SBP ≥140 mm Hg; DBP ≥90 mm Hg) hypertension by the American College of Cardiology and the American Heart Association (ACA/AHA).^3^ Although publications by the World Health Organization (WHO) (2017),^4^ American College of Obstetricians and Gynecologists (2019),^5^ and National Institute for Health and Clinical Excellence (2019)^6^ still recognize a cutoff of ≥140/90 mm Hg in pregnancy, they acknowledge an accumulating body of evidence suggesting a graded association between more moderate elevations in blood pressure and adverse pregnancy outcomes.^7^, ^8^, ^9^, ^10^, ^11^, ^12^, ^13^, ^14^ Similarly, the Hyperglycemia and Adverse Pregnancy Outcome (HAPO) study showed hyperglycemia within the GDM-range was linearly associated with adverse pregnancy outcomes, notably large birthweight (>90th centile).^15^ This has led to the recent tightening of international diagnostic criteria for GDM to capture women with milder derangements in glucose control^16^ and implementation of interventions to manage even mild GDM.^17^, ^18^, ^19^

Most studies on the association between hyperglycemia or blood pressure and pregnancy outcomes have been undertaken in high-income countries. The benefits of these screening and management approaches may not necessarily be directly translatable to other populations, particularly those in resource-poor settings, such as sub-Saharan Africa. Diabetes and hypertension are emerging as major public health problems in the region driven by changing demographics, socioeconomic, and lifestyle patterns, and it is likely that the burden of these disorders during pregnancy will mirror the rise in the general population. Indeed, the International Diabetes Federation estimates that 1 in 6 women in the African region may be affected by hyperglycemia in pregnancy,^20^ and hypertensive disorders in pregnancy are one of the most common causes of maternal death.^21^ Although blood pressure and blood glucose measurements are accepted parts of routine surveillance during antenatal care, active intervention is currently usually restricted to severe forms of hypertensive or hyperglycemic disorders. There have been only a few rigorous studies on the relationships between hypertension or diabetes, particularly in their milder forms, and pregnancy outcomes in sub-Saharan Africa.^22^, ^23^, ^24^

This study aimed to critically assess the relationship between stage 1 hypertension or fasting blood glucose levels in the GDM-range and adverse pregnancy outcomes, notably perinatal death and large birthweight, in women living in urban and peri-urban Uganda. We also examined the influence of other variables such as body mass index (BMI), maternal age, parity, and HIV status.

## Materials and Methods

### Setting

This observational cohort study was nested within a larger study to understand the burden and determinants of hyperglycemia in pregnancy in Uganda. We recruited women attending antenatal care at 5 major hospitals in urban and peri-urban areas of central Uganda between June 13, 2018, and October 31, 2019. Three of these hospitals are public facilities managed by the Uganda Ministry of Health, and 2 are private, not-for-profit hospitals managed by the Uganda Catholic Medical Services Bureau. Uganda is a low-income country in East Africa with an annual Gross Domestic Product of $643 per capita ($1.76 per day).^25^ Although great progress has been made in recent decades, the maternal mortality ratio (MMR) remains high at 368 per 100,000 live births, as does the perinatal mortality rate (PNMR) at 42 per 1000 births.^26^^,^^27^ These figures are even higher at our study sites (MMR, 401/100,000; PNMR, 67/1000; 2019 figures collated from all study sites, unpublished), presumably because of the high-risk nature of the cases referred from other centers. Similarly, although countrywide cesarean delivery rates are relatively low (11% in urban areas and 5% in rural areas),^27^ the rate in equivalent regional referral hospitals can be above 30%.^28^

### Participants

Pregnant women were eligible to participate if they were 18 years or older (age of consent) and between 24 and 28 weeks of gestation based on the date of last menstrual period or the earliest obstetrical ultrasound scan when available. Women were excluded if they met 1 or more of the following exclusion criteria: less than 18 years of age, multiple pregnancy, significant medical comorbidity (such as heart failure, renal disease, severe anemia), a known diagnosis of diabetes, inability to provide informed consent, or plans to deliver at a nonstudy facility. Women were approached in the antenatal clinic by the research team and screened for the inclusion and exclusion criteria. Women with stage 2 hypertension (≥140/90 mm Hg) or overt hyperglycemia in the DIP-range (fasting blood glucose level of ≥7.0 mmol/L based on the International Association of the Diabetes and Pregnancy Study Groups (IADPSG) and WHO criteria^29^) were referred for management by the local obstetrical team, and were excluded from subsequent analyses. Women with stage 1 hypertension (SBP, 130–139 mm Hg; DBP, 80–89 mm Hg) or hyperglycemia in the GDM-range (fasting venous blood glucose level of 5.1–6.9 mmol/L based on the IADPSG and WHO criteria^29^) were given advice and managed by the local clinical team and were included in the analyses.

At recruitment, standardized questionnaires were used to collect data about the socio-demographic background, medical (including HIV status), and reproductive history (parity, gravidity, and complications in previous pregnancies). Weight and height were measured by trained study field workers using calibrated Seca scales and stadiometers. After 30 minutes of rest, 3 seated blood pressure measurements, with 5 minutes rest between readings, were collected on the right arm using portable sphygmomanometers (OMRON-Healthcare-Co HEM-7211-E-Model-M6, Kyoto, Japan). Women were asked to fast overnight for at least 8 hours before a fasting venous blood glucose level was determined. Samples were analyzed centrally at the Medical Research Council/Uganda Virus Research Institute and London School of Hygiene and Tropical Medicine Clinical and Diagnostics Laboratory in Entebbe, Uganda, within 4 hours of collection, or stored at −80°C for subsequent analysis.

### Collection of outcome data

Maternal and neonatal outcomes were extracted from the mothers’ records at the time of delivery and recorded by midwives. These included maternal antenatal complications (hypertensive disorders of pregnancy, poly- or oligohydramnios), delivery complications (prolonged labor and ruptured uterus), mode of delivery, birthweight, and gestational age of the neonate. Hypertensive disorders of pregnancy included gestational hypertension, preeclampsia, and eclampsia. Large for gestational age was defined as a birthweight of >90th centile using the Intergrowth-21 population standards.^30^ Macrosomia and a low birthweight were defined as birthweights of >4 kg and <2.5 kg, respectively. Further data were recorded at the time of discharge from hospital and included neonatal complications (neonatal admission, hypoglycemia, jaundice) and neonatal death. Neonatal admission was defined as formal admission of the neonate to the special baby unit for observation or treatment beyond routine neonatal care.

### Definition of primary and secondary outcomes

The primary outcomes were perinatal death (stillbirth >24 weeks and neonatal death <28 days) and large birthweight for gestational age. Secondary outcomes were preterm birth (<37 weeks), cesarean delivery, and neonatal admission.

### Statistical analysis

The participants’ baseline characteristics were summarized using means, medians, standard deviations, and interquartile ranges for continuous variables where applicable. The categorical data were summarized using numbers and proportions. The sample size was calculated for the wider study and determined by an estimation of the prevalence of hyperglycemia in pregnancy in the population. The sample size was, therefore, not calculated on the basis of these perinatal outcomes. A multivariable logistic regression analysis was used to investigate the effect of stage 1 hypertension (SBP, 130–139 mm Hg; DBP, 80–89 mm Hg based on ACA/AHA criteria^3^) and hyperglycemia in the GDM-range (fasting venous blood glucose level, 5.1–6.9 mmol/L based on the IADPSG and WHO criteria^29^) on each of the primary and secondary outcomes. Confounding variables were identified by univariate analysis or widespread acceptance of their potential confounding effect. These included midgestational BMI, maternal height, maternal age, parity, HIV status, previous macrosomia, and study site. An additional exploratory analysis was undertaken to explore if our variables of interest operated in a synergistic manner with another common risk factor, obesity. Adjustments were made for the same confounding variables as the primary analysis. Results from the final adjusted models are presented as odds ratios (OR) and their 95% confidence intervals (CIs). All analyses were conducted in Stata 15.1 (College Station, TX).

### Ethical approval

This research project was approved by the research and ethics committee of the Uganda Virus Research Institute (approval GC/127/19/04/625) and the Uganda National Council for Science and Technology (approval HS2340). All participating women gave informed consent, indicated either with a signature or thumbprint. A minimal compensation was provided for the participants’ time and travel.

## Results

### Participants

This study enrolled 3852 participants. Of these, 2857 participants were included in the analysis. A total of 66 participants were excluded from analysis because of confirmed stage 2 hypertension (≥140/90 mm Hg) or overt hyperglycemia in the DIP-range (fasting blood glucose level of ≥7.0 mmol/L). The remainder of the participants (n=929) were excluded because they had incomplete laboratory and/or outcome data (Figure). Those excluded with incomplete data had similar baseline characteristics as those included in the final analysis.

**Figure:**
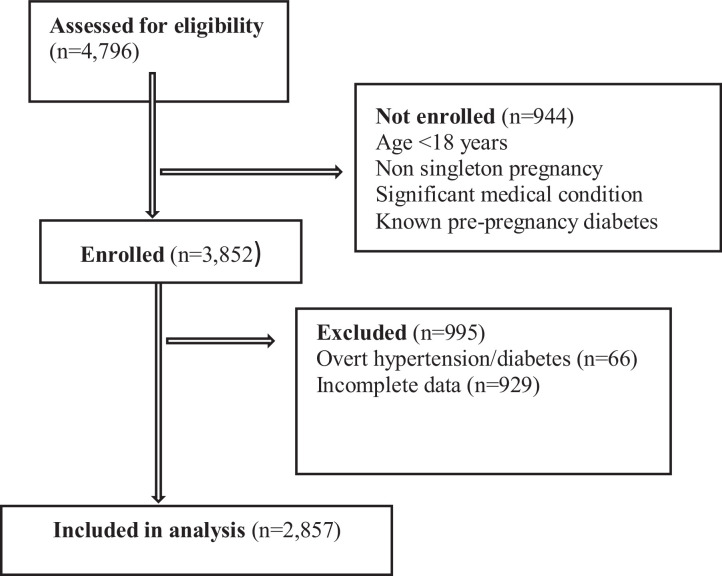
Flow chart of participant enrollment throughout the study *Milln. Moderate elevations in blood pressure or glucose and adverse pregnancy outcomes in Uganda. Am J Obstet Gynecol Glob Rep 2021*.

### Maternal characteristics

The characteristics of the participants are displayed in Table 1. All women in the study were of black ethnicity. The mean age of the participants was 26.9 years. There were 200 women with stage 1 hypertension (SBP, 130–139 mm Hg; DBP, 80–89 mm Hg) and 187 women with hyperglycemia in the GDM-range (fasting venous blood glucose level of 5.1–6.9 mmol/L); of these, 23 women had both. The mean maternal BMI was 27.7 kg/m^2^; 1087 (38.1%) women were overweight and 802 (28.1%) were obese. Nearly one-third of women were primigravid (905; 31.4%), 768 (26.9%) had 1 child, and 1184 (41.5%) had more than 1 child.

**Table 1:** Characteristics of study participants

Demographics	All participants (n=2857)
Study site, %	
Public	52.9
Private	47.1
Age (y), mean±SD	26.9±5.5
Midgestation BMI, mean±SD	27.7±6.72
Overweight (25–30 kg/m^2^), (n/N) %	(1087/2854) 38.1
Obese >30 kg/m^2^, (n/N) %	(801/2854) 28.1
Maternal height (cm), mean±SD	158.7±6.11
Stage 1 hypertension, (n/N) %	(200/2857) 7.00
SBP 130–139 mm Hg	
DBP 80–89 mm Hg	
Hyperglycemia in GDM-range, (n/N) %	(187/2857) 6.55
Fasting glucose 5.1–6.9 mmol/L	
Previous macrosomia, (n/N) %	(236/2716) 8.69
Parity, (n/N) %	
Nulliparous	(905/2857) 31.4
HIV status, (n/N) %	
Positive	(89/2857) 3.1

*BMI*, body mass index; *DBP*, diastolic blood pressure; *GDM*, gestational diabetes mellitus; *SBP*, systolic blood pressure; *SD*, standard deviation.

Milln. Moderate elevations in blood pressure or glucose and adverse pregnancy outcomes in Uganda. Am J Obstet Gynecol Glob Rep 2021.

### Maternal and neonatal outcomes

The maternal and neonatal outcomes are displayed in Table 2. One maternal death was recorded. A total of 3.7% of the participants subsequently developed hypertensive disorders of pregnancies, whereas 1.4% either had polyhydramnios or oligohydramnios. Almost one-third of the deliveries were by cesarean delivery, of which approximately three-quarters (73.2%) were recorded as “Emergency” procedures and 26.8% recorded as “Elective.”

**Table 2:** Maternal and neonatal delivery outcomes of participants

Category	(Proportion) %
Maternal outcomes	
Maternal death	(1/2857) 0.03
Antepartum complications	
Hypertensive disorder	(105/2857) 3.7
Poly- or oligohydramnios	(41/2857) 1.4
Cesarean delivery	(827/2814) 29.4
Maternal delivery complications	
Prolonged labor	(182/2857) 6.4
Ruptured uterus	(5/2857) 0.2
Neonatal outcomes	
Perinatal mortality	(74/2857) 2.6
Stillbirth (>24 wk)	(43/2857) 1.5
Neonatal death (<28 d)	(31/2814) 1.1
Mean birthweight (g), mean±SD	3275.8±546
LGA (>90th centile)	(574/2706) 22.2
Macrosomia (>4 kg)	(236/2716) 8.7
SGA (<10th centile)	(252/2706) 9.3
Low birthweight (<2.5 kg)	(226/2716) 8.3
Mean GA at delivery (wk), mean±SD	38.7±1.8
Very Preterm birth (<34 wk)	(54/2830) 1.9
Preterm birth (<37 wk)	(355/2830) 12.6
Neonatal admission	(321/2793) 11.5
Length of stay (d), median (IQR)	2 (1–4)
Neonatal complications	
Hypoglycemia	(12/2782) 0.4
Jaundice	(47/2824) 1.7

GA, gestational age; IQR, interquartile range; LGA, large for gestational age; SGA, small for gestational age; SD, standard deviation.

Milln. Moderate elevations in blood pressure or glucose and adverse pregnancy outcomes in Uganda. Am J Obstet Gynecol Glob Re*p 2021.*

There were 74 perinatal deaths, equating to a perinatal mortality rate of 26 per 1000 births. The mean birthweight of the neonates was 3275.8 g, of which 574 (22.2%) were large for gestational age on the basis of the Intergrowth-21 population standards. Preterm birth (<37 weeks) was recorded in 355 (12.6%) of the deliveries. Of the live deliveries, 321 (11.5%) were admitted to the neonatal unit with a median length of stay of 2 days. Neonatal hypoglycemia and jaundice were recorded in 0.4% and 1.7% of the cases, respectively.

### Associations with primary and secondary outcomes

Results of the multivariate analysis are displayed in Table 3. Of the 2 variables, stage 1 hypertension (adjusted OR [aOR], 2.68; 95% CI, 1.36–5.29), but not hyperglycemia in the GDM-range (aOR, 0.74; 95% CI, 0.26–2.07), was associated with an increased risk of perinatal death. Neither elevations in blood pressure nor fasting blood glucose levels were significantly associated with an increased risk of having large for gestational age babies. Among the secondary outcomes, elevated fasting blood glucose levels (aOR, 1.65; 95% CI, 1.20–2.27), but not blood pressure (aOR, 0.87; 95% CI, 0.62–1.30), was associated with an increased likelihood of cesarean delivery. Interestingly, it was maternal obesity (independent of hyperglycemia and increased blood pressure) that affected a number of the outcomes, increasing the risk of having large for gestational age babies (aOR, 2.30; 95% CI, 1.74–3.02), cesarean delivery (aOR, 2.75; 95% CI, 2.17–3.48), and neonatal admission (aOR, 1.63; 95% CI, 1.16–2.30). Other factors that were associated with adverse pregnancy outcomes included a self-reported history of macrosomia, which increased the odds of large birthweight neonates (aOR, 2.46; 95% CI, 1.84–3.27), and parity as a predictor of perinatal death (aOR, 1.30; 95% CI, 1.06–1.59).

**Table 3:** Adjusted odds ratios for associations between moderately elevated bloods glucose and blood pressure, and primary and secondary outcomes with covariates

	Primary outcomes		Secondary outcomes		
Variable	Perinatal death	Large for gestational age	Preterm birth	Cesarean delivery	Neonatal admission
Stage 1 hypertension SBP 130–139 mm Hg DBP 80–89 mm Hg	2.68 (1.36–5.29)	0.78 (0.53–1.14)	0.87 (0.62–1.30)	1.41 (0.93–2.14)	0.93 (0.60–1.44)
Hyperglycemia GDM-range 5.1–6.9 mmol/L	0.74 (0.26–2.07)	1.34 (0.94–1.90)	1.02 (0.65–1.62)	1.65 (1.20–2.27)	1.30 (0.85–1.97)
BMI 25–30 kg/m^2^^a^	0.91 (0.52–1.60)	2.01 (1.57–2.55)	0.97 (0.75–1.26)	1.74 (1.40–2.15)	1.41 (1.03–1.94)
BMI >30 kg/m^2^^a^	0.94 (0.50–1.79)	2.30 (1.74–3.02)	0.68 (0.49–0.93)	2.75 (2.17–3.48)	1.63 (1.16–2.30)
Maternal height	—	1.03 (1.01–1.04)	—	0.95 (0.94–0.97)	—
Maternal age	0.95 (0.90–1.01)	0.98 (0.96–1.01)	1.01 (0.99–1.04)	1.08 (1.05–1.10)	1.00 (0.98–1.03)
Previous macrosomia	—	2.46 (1.84–3.27)	—	—	—
Positive HIV status	2.00 (0.70–5.70)	0.79 (0.44–1.39)	—	—	—
Parity	1.30 (1.06–1.59)	0.99 (0.90–1.09)	—	0.76 (0.70–0.82)	—
Study site (private vs public)	—	1.04 (0.85–1.28)	—	—	2.96 (2.25–3.88)

*BMI*, body mass index; *DBP*, diastolic blood pressure; *GDM,* gestational diabetes mellitus; *SBP*, systolic blood pressure.*Milln. Moderate elevations in blood pressure or glucose and adverse pregnancy outcomes in Uganda. Am J Obstet Gynecol Glob Rep 2021.*

a Compared with normal BMI range (18–25 kg/m^2^).

### Combination of obesity with either stage 1 hypertension or hyperglycemia in the gestational diabetes-range

Results of this exploratory analysis are displayed in Table 4. Obesity in combination with hyperglycemia in the GDM-range appeared to increase the odds of having large birthweight for gestational age neonates, cesarean delivery, and neonatal admission. Obesity in combination with stage 1 hypertension seemed to increase the odds of perinatal death, but not preterm birth.

**Table 4:** Adjusted odds ratios for associations between obesity, in combination with either moderate elevations hyperglycemia or hypertension, and the primary and secondary outcomes

	Primary outcomes		Secondary outcomes		
Variable	Perinatal death	Large for gestational age	Preterm birth	Cesarean delivery	Neonatal admission
Stage 1 hypertension plus obesity n=102	3.23 (1.41–7.41)	1.29 (0.79–2.08)	0.68 (0.34–1.37)	0.87 (0.62–1.30)	1.09 (0.62–1.92)
Hyperglycemia GDM-range plus obesity n=92	0.34 (0.05–2.54)	1.64 (1.02–2.62)	1.10 (0.60–2.01)	2.56 (1.65–3.98)	1.92 (1.14–3.21)

*GDM*, gestational diabetes mellitus.

*Milln. Moderate elevations in blood pressure or glucose and adverse pregnancy outcomes in Uganda. Am J Obstet Gynecol Glob Rep 2021*.

## Comment

### Principal findings

Our study showed an association between stage 1 hypertension and perinatal death and adds to the growing body of evidence demonstrating an association with preterm birth. Furthermore, we demonstrated that obesity was more closely associated with large birthweight neonates, cesarean delivery, and neonatal admission than hyperglycemia in the GDM-range. This study assessed the effects of less-severe elevations in hypertension and hyperglycemia on pregnancy in Africa.

### Results in the context of other observations

In our study, the strength of the association between stage 1 hypertension and perinatal death was particularly strong. We also reported a positive trend with preterm birth, although this was not statistically significant. These findings are in accord with the well-documented physiological association between blood pressure, preeclampsia, placental underperfusion, intrauterine growth restriction, preterm delivery, and perinatal death.^31^ Many previous studies reported an interaction between stage 1 hypertension and the development of preeclampsia.^7^, ^8^, ^9^, ^10^, ^11^^,^^32^ These were performed in high-income settings and at various stages of gestation. In our study, we did not consider hypertensive disorders as a primary or secondary outcome because of the difficulties in the standardization of the diagnosis across the sites. We instead focused on more easily defined outcomes, namely perinatal death and preterm birth, which are sufficiently prevalent in our setting to warrant analysis. Other studies have demonstrated associations between stage 1 hypertension and preterm birth.^7^, ^8^, ^9^^,^^11^^,^^32^ Many of these include “indicated preterm birth,” which we presume includes induction of labor or cesarean delivery owing to preeclampsia. These interventions may be less readily available in our own setting, which may in turn contribute to the high perinatal mortality rates in line with previous reports.^21^^,^^33^ Our finding that three-quarters of cesarean deliveries were coded as “Emergency” supports this hypothesis. Only 1 other study has reported on perinatal mortality, demonstrating a positive trend, although this was not statistically significant.^11^

There was a trend of a positive association between moderate elevations in fasting blood glucose levels and large birthweight neonates. However, this did not reach the threshold for statistical significance. Indeed, elevations in fasting blood glucose levels were also associated with increased rates of cesarean delivery, presumably because of the “large babies,” although we did not have data on the indication for cesarean delivery.^34^^,^^35^ The association between fasting blood glucose levels in the GDM-range and large birthweight is in accord with the large, multinational HAPO study, which described a clear linear association between glycaemia and the risk for large birthweight neonates.^16^ Similarly, consistent with the HAPO study, we found a strong relationship between obesity and large birthweight neonates, independent of the level of glycaemia.^36^ In the HAPO study, 78% of large birthweight infants were born to mothers with normal glucose tolerance levels,^34^ whereas in our study, nearly 90% of large birthweight infants were from normoglycemic mothers. Although there are only a few studies that have explored the associations between hyperglycemia and birthweight in Africa,^24^ those that do exist also point toward BMI as a more reliable predictor of large for gestational age neonates than glycaemia.^22^^,^^37^^,^^38^

Although our study cohort was not large enough to rigorously examine the impact of multiple risk factors on the pregnancy outcomes, our exploratory analysis suggests that coexistence of our main variables of interest with other common risk factors (such as obesity) may confer additional risks. This will need confirmation in future studies.

### Clinical and research implications

This study adds to an emerging body of evidence that suggest that moderate elevations in blood pressure, below the referral threshold recommended by the WHO (blood pressure of >140/90 mm Hg), confer a significant risk for adverse perinatal outcomes. Further research is needed to understand if early identification and management of this group would be effective in reducing poor pregnancy outcomes. Furthermore, it is unclear whether such interventions would be cost-effective in areas where resources are limited, and management of overt hypertension is already lacking. One study reported that the use of aspirin can reduce the risk of preeclampsia in women with stage 1 hypertension, but this involved a secondary analysis of data from a study performed almost 3 decades ago in a very different setting.^9^ The clear association with perinatal death in the absence of a significant association with preterm birth also warrants further investigation. Indeed, a multicenter trial is already underway to assess whether planned preterm delivery (34–37 weeks’ gestation), compared with expectant management, can reduce adverse pregnancy outcomes in mothers with preeclampsia.^39^ These types of studies will help to inform wider strategies to reduce the unacceptably high rates of stillbirths and neonatal death in this setting, an area finally attracting much needed attention.^40^

Our finding that obesity is a stronger predictor of large birthweight neonates and cesarean delivery than moderate elevations in hyperglycemia, underscores the contribution of other (nonglucose) physiological determinants of fetal growth. The finding that mothers’ self-report of previous macrosomia also predicted large birthweight neonates, as in other settings,^41^^,^^42^ suggests a clustering of risk. The identification of parameters such as obesity and a history of macrosomia provides the potential for cost-effective screening and/or intervention opportunities, particularly in settings were resources are limited and the measurement of blood glucose levels is not currently feasible.

### Strengths and limitations

To our knowledge, this is the largest prospective study to critically investigate the associations between moderate elevations in blood pressure or blood glucose and pregnancy outcomes in Africa. We used standardized international definitions (stage 1 hypertension,^3^ and fasting hyperglycemia in the GDM-range^29^^,^^43^) to aid in our comparison with wider literature. We made efforts to ensure that it was as representative as possible by recruiting from both public and private facilities. We made use of a rigid study protocol to collect detailed data, utilized a high-quality central laboratory for sample analyses, and employed up-to-date diagnostic criteria.

In our study, women with moderate elevations in fasting blood glucose levels and who were therefore diagnosed with GDM, were managed by their clinicians rather than through a study protocol; the intensity of treatment was therefore likely to be variable and not under study control, which may have influenced the outcomes. However, women with moderate elevations in blood pressure were not referred for further management, as per national guidance. For some outcomes (such as preeclampsia and poly- and oligohydramnios), we relied on healthcare records rather than active investigation by the study team. We did not have data regarding previous operative deliveries and could therefore not determine whether it was a primary cesarean delivery or not, as in the HAPO study.^15^ Although serial blood pressure recordings were taken throughout the antenatal period, we concluded that the heterogeneity of conditions and devices used was not conducive of reliable results. The loss to follow-up owing to women delivering elsewhere may have introduced bias in the outcomes reported, although the baseline characteristics of those included and excluded in the analysis were similar. The study was performed in urban and peri-urban central Uganda, where women regularly attend antenatal clinics. This may reduce the generalizability to rural populations or tertiary referral hospitals with more selected specialist cases. This is highlighted by the relatively low MMR and perinatal mortality rate in our study than the overall referral hospital figures and those of other regions in the country.

### Conclusion

Our study in Uganda indicates that subtle elevations in blood pressure, and to a lesser extent blood glucose levels, during pregnancy increase the risk for adverse outcomes in the mother and offspring. These data also highlight the importance of obesity as an independent driver of these risks. Simple and cost-effective interventions to screen and manage these risk factors, particularly obesity and blood pressure, might lead to significant reductions in adverse outcomes in this population.
